# A quick and low-cost PCR-based assay for *Candida* spp. identification in positive blood culture bottles

**DOI:** 10.1186/1471-2334-13-467

**Published:** 2013-10-07

**Authors:** Hemílio Xafranski, Analy SA Melo, Antonia M Machado, Marcelo RS Briones, Arnaldo L Colombo

**Affiliations:** 1 Laboratório Especial de Micologia, Departamento de Medicina, Universidade Federal de São Paulo, Rua Pedro de Toledo 669 5 andar, São Paulo, SP, Brazil; 2 Laboratório de Genômica Evolutiva e Biocomplexidade, Departamento de Microbiologia, Imunologia e Parasitologia, Universidade Federal de São Paulo, Rua Pedro de Toledo 669 4 andar, São Paulo, SP, Brazil; 3 Laboratório Central do Hospital São Paulo, Rua Napoleão de Barros 715 2 andar, São Paulo, SP, Brazil

**Keywords:** Candidemia, Molecular diagnosis, ITS region sequencing, Fungal DNA extraction

## Abstract

**Background:**

Differences in the susceptibility of *Candida* species to antifungal drugs make identification to the species level important for clinical management of candidemia. Molecular tests are not yet standardized or available in most clinical laboratories, although such tests can reduce the time required for species identification, as compared to the conventional culture-based methods. To decrease laboratory costs and improve diagnostic accuracy, different molecular methods have been proposed, including DNA extraction protocols to produce pure DNA free of PCR inhibitors. The objective of this study was to validate a new format of molecular method, based on the internal transcribed spacer (ITS) of the rDNA gene amplification followed by sequencing, to identify common and cryptic *Candida* species causing candidemia by analyzing DNA in blood culture bottles positive for yeasts.

**Methods:**

For DNA extraction, an “in-house” protocol based on organic solvent extraction was tested. Additional steps of liquid nitrogen incubation followed by mechanical disruption ensured complete cell lysis, and highly pure DNA. One hundred sixty blood culture bottles positive for yeasts were processed. PCR assays amplified the ITS region. The DNA fragments of 152 samples were sequenced and these sequences were identified using the GenBank database (NCBI). Molecular yeast identification was compared to results attained by conventional method.

**Results:**

The organic solvent extraction protocol showed high reproducibility in regards to DNA quantity, as well as high PCR sensitivity (10 pg of *C. albicans* DNA and 95% amplification on PCR). The identification of species at the molecular level showed 97% concordance with the conventional culturing method. The molecular method tested in the present study also allowed identification of species not commonly implicated in human infections.

**Conclusions:**

This study demonstrated that our molecular method presents significant advantages over the conventional yeast culture identification method by providing accurate results within 24 hours, in contrast to at least 72 hours required by the automated conventional culture method. Additionally, our molecular method allowed the identification of mixed infections, as well as infections due to emergent fungal pathogens. This economical DNA extraction method developed in our laboratory provided high-quality DNA and 60% cost savings compared to commercial methods.

## Background

Nosocomial candidemia remains a frequent cause of morbidity and mortality, mainly among immunocompromised and intensive care (ICU) patients [1-3]. Mortality remains high, mainly due to the severity of underlying conditions associated with candidemia, as well as limitations in the conventional tools available for early diagnosis of this infection [4-6].

The clinical signs of sepsis due to candidemia are nonspecific, and diagnosis generally relies on conventional blood culture, which is often delayed and presents sensitivity lower than 70% [7,8]. Early diagnosis of this infection is essential for proper antifungal therapy, and delays of more than 12 hours in the administration of antifungal agents may substantially increase mortality [9].

PCR methods for the diagnosis of *Candida* bloodstream infections are not yet internationally standardized, and divergent results may be obtained when different parameters are used to perform the test. Indeed, there are still controversies regarding the selection of biological material to be tested (whole blood, plasma or serum), various protocols for DNA extraction, the gene target to be analyzed, the PCR assay used (conventional, nested, PCR-RFLP or real time), the number of cycles, the detection limit and the parameters used for sequences analysis [10-16]. In the absence of commercial systems largely validated in multicenter studies, “in-house” PCR based methods remain used worldwide for the diagnosis of candidemia.

In the present study, we validated a new format of molecular method based on ITS-PCR followed by sequencing to identify common and cryptic *Candida* species causing candidemia by analyzing fungal DNA in yeast-positive blood culture bottles using a rapid and accurate molecular method. We also standardized a low-cost method for DNA extraction, developed “in-house”, that yielded highly purified DNA and provided 60% cost savings compared to the commercial methods.

## Results and discussion

### Fungal DNA extraction

DNA was purified from blood samples using an extraction method described below that yielded high-quality DNA (high purity with respect to proteins and salt, optical density (OD) 260/280 and 260/230 with rate values ≥ 1.8) and decreased the effect of PCR inhibitors such as hemoglobin and some components of the culture medium.

It has been well documented that the fungal cell wall is an important obstacle to nucleic acid extraction and that the effectiveness of the cell lysis and DNA extraction highly influences the sensitivity of PCR assays [17,18].

Our methodology included two important cell lysis steps: freezing the sample with liquid nitrogen and agitation with glass beads. Both procedures have been successfully used by other authors to attain fungal cell lysis and DNA extraction [18-20]. We used the Brazol® reagent (guanidine thiocyanate), an inexpensive compound that is able to disrupt cell membranes, fragment chromosomes, and prevent DNA from being trapped in clinical sample residues, reducing the activity of PCR inhibitors [21].

Our extraction protocol was designed to extract both intracellular and extracellular DNA from the sample [22]. This protocol was standardized by testing negative blood culture bottles and *C. albicans*-spiked blood culture as negative and positive controls, respectively (data not shown).

This “in-house”-developed DNA extraction method provided approximately 60% cost savings compared to commercial methods.

### PCR for fungal ITS amplification and sequencing

**Figure 1:**
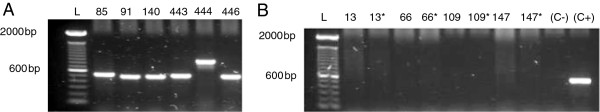
**Amplification of DNA extracted from positive blood culture bottles for yeasts.** One percent agarose gels presenting the PCR product from: **A)** Six positive samples amplified through our molecular method, yielding fragments of 400-700 bp; **B)** Four positive samples representative of eight samples that could not be amplified by our method. These samples were spiked with DNA extracted from *C. albicans* ATCC 24433. None of the samples could be amplified, in spite of the addition of *C. albicans* DNA. * Samples spiked with *C. albicans* ATCC 24433 DNA; (C-) Negative control (PCR grade water); (C+) Positive control (*C. albicans* ATCC 24433 DNA).

Of 160 DNA samples extracted from blood culture bottles positive for yeasts, successful amplification of the ITS was achieved in 152 samples (95%) using our PCR methodology. Our method yielded fragments of 400-700 bp (see Figure 1A).

The ITS 1, ITS 2 and 5.8S regions of rDNA have already been well documented as a reliable target for amplification and sequencing, useful for discriminating between medically important yeasts [10,23]. Within the ITS rDNA region, we selected the universal fungal-specific primers ITS1 and ITS4 because our preliminary studies demonstrated that these primers were more specific for fungal DNA, presenting lower levels of cross-reaction with human DNA than other primers. We also used a high hybridization temperature (58°C), which allowed the specific amplification of fungal DNA. The ITS sequences were obtained using the same primers used in the PCR assay. Results from BLASTn that provided an e-value less than 10^-5^ and a maximum identity higher than 98% were considered reliable for identification of *Candida* species.

Only 8 infection samples could not be amplified by our PCR method, as follows: 2 samples of *C. albicans*, 2 samples of *C. parapsilosis*, 2 samples of *C. tropicalis*, 1 sample of *C. pelliculosa* (*Pichia anomala)* and 1 mixed infection sample of *C. parapsilosis* and *C. guilliermondii*. To check for the presence of PCR inhibitors, *C. albicans* ATCC 24433 genomic DNA was added to each sample prior to the PCR assay. These samples could not be amplified despite the addition of *C. albicans* DNA (Figure 1B). These findings suggest that the cause of negative PCR results was neither the detection limit of yeast DNA for the test nor insufficient DNA quantities in the sample. Rather, it was caused by the presence of PCR inhibitors that were not removed by Brazol during DNA extraction or by the addition of bovine serum albumin (BSA) to the PCR assay.

The presence of PCR inhibitors such as hemoglobin, lactoferrin and sodium polyanetholesulfonate from erythrocytes, leukocytes and blood culture media, respectively, may explain false negative results generated along DNA amplification from clinical samples [24]. The inhibitory effect of hemoglobin and lactoferrin seems to be related to the ability of these proteins to release iron ions. The inhibitory effect of iron ions appears to act directly on the DNA polymerase by competing with the template. Lactoferrin also appears to interact with nucleic acids. Al-Soud and Radstrom [24] tested several amplification facilitators in the presence of hemoglobin and lactoferrin. BSA was the most efficient compound in reducing inhibition of amplification, due to its binding efficiency. Consequently, BSA is often used to increase the sensitivity of PCR amplification from clinical samples [24-26].

With the exception of the 8 samples that were not amplified in this study, the addition of BSA to the PCR assay allowed us to amplify most of the samples.

### Comparison between the conventional culture-based *Candida* spp. identification method and our molecular method

The identification of *Candida* spp. obtained by the conventional culture-based and molecular methods is shown on Table 1. The results of the genotypic identification method correlated highly with the conventional culture-based identification method (97%). It is important to stress that discordant identifications corresponded to cryptic species (e.g., the *C. haemulonii, C. intermedia* and *C. parapsilosis* species complex). These species were not accurately identified by the conventional method but were identified by the present PCR- and sequencing-based method. In addition, our molecular method was able to identify one episode of mixed infection (*C. albicans* and *C. glabrata*), while the conventional culture-based method recognized only *C. albicans* in the mixed infection. This difference between assays is most likely due to the unviability of *C. glabrata* cells for culture, rendering them undetectable by the conventional culture-based method. However, the persistence of detectable DNA in the sample allowed for successful identification by the molecular method. In contrast, one episode of mixed infection caused by *C. glabrata* and *C. albicans* was recognized by the culture method, while our molecular method identified only *C. albicans.* This anomaly was most likely the result of low burden of *C. glabrata* in the sample, especially in the low volume of extracted DNA used for PCR assay. As noted by Pfeiffer et al. [22], despite the high sensitivity of conventional PCR, a minimum of 10 CFU/ml is generally required to detect fungal DNA.

**Table 1:** Comparison of phenotypic and genotypic identification of yeasts obtained from positive blood culture bottles

**Phenotypic identification**		**Genotypic identification**	
**Species**	**Number of**	**Species**	**Number of**
	**identified**		**identified**
	**samples**		**samples**
***C. albicans***	41	*C. albicans*	40
		*C. albicans + C. glabrata*	1
***C. glabrata***	28	*C. glabrata*	28
***C. guilliermondii***	4	*Meyerozyma guilliermondii*	2
		*Clavispora lusitaniae*	1
		*C. intermedia*	1
***C. krusei***	15	*Issatchenkia orientalis*	15
***C. lusitaniae***	1	*Clavispora lusitaniae*	1
***C. norvegensis***	1	*Pichia norvegensis*	1
***C. parapsilosis (sensu lato)***	34	*C. parapsilosis (sensu stricto)*	27
		*C. orthopsilosis*	7
***C. pelliculosa***	1	*C. haemulonii*	1
***C. tropicalis***	26	*C. tropicalis*	26
***C. glabrata + C. albicans***	1	*C. albicans*	1
**Total**	**152**		**152**

*Issatchenkia orientalis*: teleomorph of *C. krusei*; *Clavispora lusitaniae*: teleomorph of *C. lusitaniae*; *Meyerozyma guilliermondii*: teleomorph of *C. guilliermondii*; *Pichia norvegensis*: teleomorph of *C. norvegensis*.

In contrast with methodologies that adopt real-time PCR and/or nested PCR [27,28], our method is not limited to the use of a small number of probes or primers. Our method also provides accurate identification of emergent pathogens and novel sequence variants for which species-specific primers or probes have not yet been validated.

Through this molecular method, after the bottles with positive cultures were detected, we were able to extract DNA from the blood cultures and accurately identify fungal species within 24 hours, while the conventional non-automated method would take at least 72-96 hours [29]. Morrell et al. [6] has previously demonstrated that a 12-hour delay in the initiation of antifungal treatment is sufficient time to double mortality in patients with candidemia. By using the ITS-PCR and sequencing method developed in this study, it is possible to reduce the time required to identify the causal yeast species in an infection by 48 hours compared to the conventional culture-based method.

### Performance of ITS-PCR and sequencing compared to other molecular methods

The ITS-PCR and sequencing method validated in our laboratory showed low-cost, reliable and accurate *Candida* species identification; it yielded accurate final identification in 152 out of 160 (95%) blood cultures tested. The reproducibility of the assay was 100% as checked by a pilot study testing negative blood culture bottles spiked with cultured *Candida* spp. cells (data not shown). Our molecular method allowed the identification of rare and cryptic species, such as *C. intermedia, C. haemulonii* and *C. orthopsilosis* (the last one is a species of the *C. parapsilosis* complex). Identification was accomplished with tools and techniques that are accessible to routine laboratories, including a low-cost “in-house” DNA extraction protocol and an automatic sequencer that uses the Sanger method.

A large variety of PCR-based methods has been applied to identify candidemia in blood culture bottles, but most of these methods used specific primers and probes that may potentially limit the number of species that can be identified. Shin et al*.* has described a clinically useful PCR method, followed by identification with species-specific probes that could detect only the five most common *Candida* species in 73 blood culture bottles from 31 candidemic patients [29]. These probes could not identify cryptic species of the *C. parapsilosis* complex, *C. orthopsilosis* and *C. metapsilosis*.

Other authors have developed semi-nested or multiplex PCR methods involving the amplification of yeast rDNA regions, resulting in fragments of different sizes for species identification. These methods were not able to differentiate between all the species tested, including the rare and cryptic species, especially the ones classified in species complex, such as *C. parapsilosis* and *C. guilliermondii*[10,30-32].

Other PCR-based methods followed by DNA sequencing have been successfully developed. However, these studies have used commercial methods for DNA extraction, which makes this process more expensive than our “in-house”-developed DNA extraction method [33,34]. Additionally, Quiles-Melero et al*.* used a PCR-based methodology coupled with pyrosequencing [34]. The authors reported accurate *Candida* spp. identification in all 47 blood culture bottles tested, yielding 100% correlation with the conventional method. However, pyrosequencing methodology is not available for candidemia diagnosis in most medical centers.

## Conclusions

Several studies have been published describing the use of conventional, nested or real-time PCR in positive blood culture bottles to identify yeast species; however, no molecular methodology has been broadly adopted for the identification of yeast species in modern clinical laboratories. Even direct PCR of blood from patients with suspected candidemia would provide more rapid species identification. Unfortunately, this methodology yields low positivity due to the low fungal burdens usually present in whole blood samples [15,22,35,36].

In the present study, we described and validated a new format for a molecular-based method of yeast identification. This method is based on ITS-PCR followed by sequencing to identify common, rare and cryptic *Candida* species that cause candidemia and are present in yeast-positive blood culture bottles. This molecular method for the identification of *Candida* spp. had a significant advantage over the conventional culture-based identification method by providing accurate results within 24 hours, in contrast to at least 72 hours required by the automated conventional culture. The molecular method developed in this study additionally provided identification of mixed infections as well as infections due to emergent fungal pathogens. Furthermore, the low-cost DNA extraction method developed in our laboratory provided high-quality DNA and 60% cost-savings compared to commercial methods.

## Methods

### Selection of biological samples and controls for PCR

We selected a total of 160 yeast-positive blood culture bottles among 405 bottles of the BACTEC system yielding yeasts that were sequentially processed in the clinical laboratory of the Hospital São Paulo, Universidade Federal de São Paulo, between October 2008 and February 2012. Immediately after confirmation of yeast growth in blood cultures, aliquots from each bottle were taken for conventional fungal culture and microorganism identification. Finally, aliquots of 3-4 mL of each bottle processed during the period were stored at -20°C for further PCR analysis.

The selection of samples for further PCR assays aimed to include cultures representative of most clinically relevant species of *Candida* based on the initial phenotypic identification obtained in our clinical laboratory of the yeasts growing in blood cultures. Based on the initial identification of yeast colonies generated by the ID 32 system and complemented by the micromorphology findings, 152 cultures were studied. These cultures included the most common species that cause candidemia: *C. albicans* (41/27.1%), *C. parapsilosis* (34/22.5%), *C. tropicalis* (26/17.2%), *C. glabrata* (28/18.5%), *C. krusei* (15/9.9%), and *C. guilliermondii* (4/2.6%). In addition, we selected 3 cases representative of candidemic episodes due to unusual pathogens as identified by the ID-32 system: *C. pelliculosa* (1/0.7%), *C. lusitaniae* (1/0.7%) and *C. norvegensis* (1/0.7%). We also included one case of mixed infection caused by *C. albicans* and *C. glabrata* (1/0.7%).

Finally, as negative controls for the *Candida* PCR assay, we included DNA samples from blood cultures representative of sepsis due to Gram-positive (10 samples) and Gram-negative bacteria (10 samples), as well as 10 negative blood cultures. Genomic DNA extracted from the reference strain *Candida albicans* ATCC 24433 was used as a positive control.

### DNA extraction from blood culture bottles

DNA was extracted from all samples according to the method adapted from Sandhu et al*.*[21]. For this procedure, 300 μL of each sample and 200 μL of glass beads were added to a 1.5 mL microtube, incubated in liquid nitrogen for 2 minutes and vigorously shaken for 3 cycles of 45 seconds in a FastPrep homogenizer at speed 5.0. After cooling the tubes on ice, 600 μL of room temperature Brazol was added and the tubes were vortexed. Subsequently, 130 μL of cold chloroform was added, and the tubes were shaken again. The samples were then centrifuged at 20,000 × *g*, at 8°C for 12 minutes and the supernatants were transferred to new tubes containing 500 μL of cold absolute ethanol. The tubes were shaken by hand, and the samples were again centrifuged at 20,000 × *g* at 8°C for 12 minutes. The pellets were washed twice with 500 μL of 70% ethanol, dried and resuspended in 30 μL of Milli-Q water. Each sample was tested for DNA quantity, and protein and salt contamination via reading at OD 260/280 and 260/230, respectively. DNA was re-extracted from samples that had ratios <1.8. Purified DNA was stored at -20°C until further procedures were performed.

### PCR assay for rDNA ITS region amplification

To amplify the rDNA ITS1, 5.8 S, and ITS2 regions, we performed PCR assays with the primer pair ITS1 and ITS4 at 10 pmol/μL, in a final volume of 25 μL containing: 12.5 μL of PCR MasterMix (Promega, Madison, WI – USA), 2 μL ITS1 primer (forward), 2 μL of ITS4 primer (reverse), 2.5 μL of 5% BSA, 4 μL of Milli-Q water and 2 μL of DNA sample. The thermal cycling protocol used was as follows: an initial denaturation phase of 5 minutes at 94°C, followed by 35 cycles of 94°C for 1 minute, 58°C for 1 minute, 72°C for 2 minutes, and finally an additional extension phase at 72°C for 10 minutes. Each PCR batch included one reaction containing Milli-Q water as a negative control and one reaction containing DNA extracted from *C. albicans* ATCC 24433 culture as a positive control.

The PCR samples were submitted to agarose gel electrophoresis to verify DNA amplification. When two or more amplicons were visualized on a gel, each band was purified using a GFX kit (GE Healthcare, UK), and each fragment was sequenced independently.

### ITS sequencing and yeast species identification

The sequencing reaction was performed through the addition of 2 μL of diluted amplicon (1:10 to intense bands, 1:5 to weak bands) to a reaction mix containing 2 μL of BigDye Terminator (Applied Biosystems, USA), 4 μL of sequencing buffer (Applied Biosystems, USA), 4 μL of Milli-Q water and 3 μL of 2 pmol/μL primer, in a total reaction volume of 15 μL. The reactions were submitted to 25 thermal cycles as follows: 95°C for 20 seconds, 50°C for 15 seconds and 60°C for 1 minute. The reactions were precipitated through the addition of 2 μL of 3 M sodium acetate/well, 2 μL of 125 mM EDTA/well and 55 μL of ethanol per well and then denatured through the addition of 15 μL of formamide HiDi (Applied Biosystems, USA) and incubation at 94°C for 4 minutes. Finally, the samples were rapidly cooled on ice before the reactions were run on the automatic sequencer.

The amplicons were sequenced using the Sanger methodology in a MicroAmp – Optical 96-well reaction plate (Applied Biosystems, USA) and the automatic sequencer ABI 3130 (Applied Biosystems, USA). At least 2 reads were generated for each sample.

The chromatograms generated by each sample were assembled into contigs and edited using the software programs Phred Phrap and Consed, respectively [37-40]. Alignments with <100 errors per 10 Kb were considered valid. The sequences were then compared to the NCBI genomic database using the BLASTn tool. Species identifications were accepted when the BLASTn values were e-value < 10^-5^ and identity ≥ 98% [41].

### Performance evaluation of the PCR assay

Species identification generated by our PCR method was compared to results obtained by the conventional culture-based identification method.

Putative assays with no PCR amplification were checked for the presence of PCR inhibitors through the addition of *C. albicans* ATCC genomic DNA.

### Ethics statement

The protocol was approved by the Ethics Committee of the Federal University of Sao Paulo (n. 1414/09).

## Competing interests

The authors declare that they have no competing interests.

## Authors’ contributions

HX carried out the molecular techniques, participated in the sequence alignment and drafted the manuscript. ASAM adapted and standardized the study methodology, assisted the data analysis and drafted the manuscript. AMM coordinated the sample selection for this study. MRSB contributed to the study design. ALC conceived and coordinated the study design and the draft of the manuscript. All authors read and approved the final manuscript.

## Pre-publication history

The pre-publication history for this paper can be accessed here:

http://www.biomedcentral.com/1471-2334/13/467/prepub
